# Lung complications are common in intensive care treated patients with pelvis fractures: a retrospective cohort study

**DOI:** 10.1186/s13049-016-0244-1

**Published:** 2016-04-19

**Authors:** Joakim Engström, Henrik Reinius, Jennie Ström, Monica Frick Bergström, Ing-Marie Larsson, Anders Larsson, Tomas Borg

**Affiliations:** Anesthesiology and Intensive Care, Department of Surgical Sciences, Uppsala University, SE-751 85 Uppsala, Sweden; Orthopedics, Department of Surgical Sciences, Uppsala University, SE-751 85 Uppsala, Sweden

**Keywords:** Acute hypoxic failure, Acute respiratory distress syndrome, Traumatic pelvis fracture, Intensive care units

## Abstract

**Background:**

The incidence of severe respiratory complications in patients with pelvis fractures needing intensive care have not previously been studied. Therefore, the aims of this registry study were to 1) determine the number of ICU patients with pelvis fractures who had severe respiratory complications 2) whether the surgical intervention in these patients is associated with the pulmonary condition and 3) whether there is an association between lung complications and mortality. We hypothesized that acute hypoxic failure (AHF) and acute respiratory distress syndrome (ARDS) 1) are common in ICU treated patients with pelvis fractures, 2) are not related to the reconstructive surgery, or to 3) to mortality.

**Methods:**

All patients in the database cohort (*n* = 112), scheduled for surgical stabilization of pelvis ring and/or acetabulum fractures, admitted to the general ICU at Uppsala University Hospital between 2007 and 2014 for intensive care were included.

**Results:**

The incidence of AHF/ARDS was 67 % (75/112 patients), i.e., the percentage of patients that at any period during the ICU stay fulfilled the AHF/ARDS criteria. The incidence of AHF was 44 % and incidence of ARDS was 23 %. The patients with AHF/ARDS had more lung contusions and pneumonia than the patients without AHF/ARDS. Overall, there were no significant changes in oxygenation variables associated with surgery. However, 23 patients with pre-operative normal lung status developed AHF/ARDS in relation to the surgical procedure, whereas 12 patients with AHF/ARDS normalized their lung condition. The patients who developed AHF/ARDS had a higher incidence of lung contusion (*P* = 0.04) and the surgical stabilization was performed earlier (5 versus 10 days) in these patients (*P* = 0.03).

**Conclusions:**

We found that the incidence of respiratory failure in ICU treated patients with pelvis fractures was high, that the procedure around surgical stabilization seems to be associated with a worsening in the respiratory function in patients with lung contusion, and that mortality was low and was probably not related to the respiratory condition.

**Trial registration:**

Study was registered at ISRCTN.org number, ISRCTN10335587.

## Background

Respiratory complications are common after major trauma and one of the most serious forms is acute respiratory distress syndrome (ARDS) with an incidence of 12–25 % [1, 2]. ARDS is defined as an acute inflammatory pulmonary condition with hypoxemia combined with bilateral lung infiltrates seen with computed tomography or x-ray. The cause should be an inciting insult such as sepsis, major surgery or trauma [3]. Risk factors for developing ARDS in trauma are injury severity score (ISS), pulmonary contusions [4, 5], blunt injury mechanism, flail chest [5] and massive transfusion [5, 6]. It is well recognized that pelvis fractures are associated with respiratory failure including ARDS [5, 7, 8]. Theoretically, respiratory failure could be aggravated by a “second hit” such as an inflammatory response induced by a surgical procedure. Therefore, surgical fixations in patients with pelvis fractures have sometimes been postponed in patients due to this reason. There are clear indications that early fixation reduces respiratory complications in patients both with femur- and pelvis fractures [9, 10]. However, whether the surgical procedure per se is associated with deterioration in lung function has to our knowledge, not been studied in patients with pelvis fractures. Moreover, although it is well known that intensive care treated patients with pelvis fractures often have respiratory complications, it has not been studied whether these specifically are associated with worse outcome [10]. Indeed, morbidity and mortality in ARDS caused by trauma is much lower than for other underlying conditions. Thus, mortality in a mixed intensive care unit (ICU) population with ARDS is 30 – 45 % [1, 11–13], but in trauma patients with ARDS the mortality is 9 – 25 % [2, 14]. In addition, the incidence of severe respiratory complications in a European cohort of patients with pelvis fractures needing intensive care have not, what we are aware of, been studied. Therefore the pre-specified aims of this registry study were to 1) assess the incidence of severe respiratory complications, i.e., ARDS or severe hypoxemic failure (AHF, as defined as ARDS without radiological criteria or when no radiologic examination was performed) in patients with pelvis fractures in our ICU (the hospital is a referral center for pelvis fractures), 2) whether the surgical intervention in these patients is associated with worsening of the pulmonary condition and 3) whether the lung complications is associated with mortality. The hypotheses were that ARDS/AHF 1) is common in ICU treated patients with pelvis fractures, 2) will not be related to surgical stabilization, or 3) to mortality.

## Methods

The study was approved by the local ethics committee at Uppsala University (2006/140), Uppsala, Sweden and the study was registered at ISRCTN.org number, ISRCTN10335587. Informed consent was obtained from the patient before inclusion in the database.

Data was used from a cohort of 669 patients admitted to the Uppsala University Hospital scheduled for surgical stabilization of pelvis ring and/or acetabulum fractures. Except the patients in the local region of the Uppsala University Hospital, 30 additional hospitals referred patients after providing primary care.

### Patient selection

All patients in the database cohort, admitted to the general ICU at Uppsala University Hospital between 2007 and 2014 for intensive care treatment/monitoring were prospectively included. Exclusion criteria were; 1. Not admitted to the ICU. 2. No arterial line present during the ICU stay. 3. Less than 18 years of age. 4. Pregnancy.

**Fig. 1:**
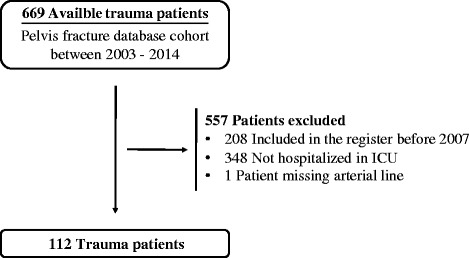
Patients included from the Pelvis fracture database cohort

One hundred and twelve patients were eligible for inclusion in the study (Fig. 1).

Demographical/medical data, ICU/hospital stay, ICU/hospital mortality and 60-day mortality were retrospectively recorded from the database. From the medical charts, data was collected and the following scores were calculated: Simplified acute physiology score (SAPS 3), Sequential organ failure assessment score (SOFA), Injury severity score (ISS), New injury severity score (NISS), Abbreviated injury scale (AIS) and Glasgow coma scale (GCS). In addition, from similar sources we registered the incidence of thoracic injury, time between injury and surgical intervention, duration of surgery and perioperative blood loss. The number of transfusions of red blood cells; fresh frozen plasma and platelets was recorded from the ICU and anesthesia charts as well as the amount of synthetic colloids and crystalloids administered between date of injury until discharge from ICU. Moreover, ventilator data (i.e., tidal volumes, airway pressures and inspired oxygen concentrations) and arterial blood gas values (PaO_2_, PaCO_2_, pH and base excess (ABL 800 Flex, Radiometer, Brondby, Denmark)) were collected from the medical charts.

In all patients, low-molecular weight heparin was administered subcutaneously as prophylaxis against venous thrombosis for a minimum of 10 days after surgery, and prolonged for patients not mobilized by that time. Systemic prophylactic antibiotics were given perioperative for a minimum of 24 h.

### AHF/ARDS

The Berlin definition of ARDS [15] was used in this study. AHF was defined according to the ARDS definition without the radiologic criterion. Therefore, patients with oxygenation deficits according to the Berlin definition were included into two groups; They who fulfilled the radiological criterion were allocated to the ARDS group, whereas those who did not fulfill this criterion were allocated to the AHF group.

All patients’ radiological chest images (both standard radiograms and computed tomography (CT)) were downloaded from the hospital radiological system, Vue Motion® 12.0 (Carestream Health Inc., Rochester, NY, US). Two chest radiological examinations were selected for analysis, one pre- and one post-operative. The chest images used for the analysis were obtained within 2 days before and within 2 days after surgery, respectively. Two arterial oxygen tension (PaO_2_)/Inspired oxygen fraction (FiO_2_) ratios were used; the lowest values within ± 12 h from the time point when the chest images were obtained. In the case when no chest radiological examination was performed, the two PaO_2_/FiO_2_ ratios used in the calculation of AHF were the lowest values at 48 ± 12 h before and 48 ± 12 h after surgery, respectively. Single outlying PaO_2_/FiO_2_ ratios values were excluded.

Two experienced consultant intensivists analyzed independently all radiological chest images to determine whether the radiological criteria of ARDS were fulfilled. The examiners were not aware of the oxygenation data or whether the images were taken before of after the surgical procedure. For the images where there was a disagreement, the images were reexamined in order to achieve a consensus.

### Statistics

For the statistical analyses, the SPSS 23.0 for Windows/Mac OS X statistical program (IBM Corp., Armonk, NY, USA) was used. One-way ANOVA with a post hoc test (Tukey) was used for the analysis of the differences among patients with and without AHF and ARDS. Independent *t*-test was used in for the analysis of the difference among patients with pre-operative normal lung status who developed AHF/ARDS in relation to the surgical procedure and patients with AHF/ARDS who normalized their lung condition. *P* < .05 was considered a priori as statistically significant. All values are mean ± standard deviation (SD).

## Results

One hundred and twelve patients, 29 women and 83 men were enrolled in this study. General characteristics are presented in Table 1.

**Table 1:** Characteristics for all patients and patients with and without acute hypoxic failure or acute respiratory distress syndrome

Variable	All patients (*n* =112)	Patients with AHF (*n* = 49 (44 %))	Patients with ARDS (*n* = 26 (23 %))	Patients with no AHF/ARDS (*n* = 37 (33 %))	*P* value
Age (yrs)	48 ± 18	48 ± 19	51 ± 19	46 ± 18	0.56
Female sex, no. (%)	29 (26)	13 (27)	5 (19)	11 (30)	0.65
Injury severity score (ISS)	29 ± 12	30 ± 13	29 ± 11	26 ± 11	0.27
New Injury severity score (NISS)	32 ± 12	33 ± 13	30 ± 11	30 ± 12	0.31
Simplified Acute Physiology Score (SAPS 3)	45 ± 10	45 ± 10	45 ± 10	45 ± 10	0.80
Sequential Organ Failure Assessment score (SOFA)	7 ± 4	9 ± 4^a^	7 ± 3a	5 ± 3	<0.0001
Glasgow Coma Scale (GCS)	13 ± 3	11 ± 4^a, b^	14 ± 2	14 ± 3	<0.001
Blunt injury mechanism, no. (%)	112 (100 %)	49 (100)	26 (100)	37 (100)	n.a.
Pulmonary contusion, no. (%)	27 (24)	12 (25)	11 (42)^a^	4 (11)	0.02
Pneumothorax before admission, no. (%)	43 (38)	20 (41)	12 (46)	11 (30)	0.38
Pulmonary embolism, no. (%)	7 (6)	4 (8)	2 (8)	1 (3)	0.56
Pneumonia, no. (%)	16 (14)	8 (16)	7 (27)^a^	1 (3)	0.02
Time to surgery after injury, d	6 ± 4	7 ± 4	6 ± 3	5 ± 4	0.25
Total time of surgery, min	233 ± 152	226 ± 132	237 ± 175	241 ± 163	0.9
Perioperative blood loss, mL	1085 ± 1419	834 ± 831	1573 ± 2330	1034 ± 1025	0.44
PaO2/FiO2 ratio pre-operative	28 ± 15	23 ± 10^a^	21 ± 12^a^	40 ± 16	<0.0001
PaO2/FiO2 ratio post-operative	27 ± 13	26 ± 10^a^	22 ± 6^a^	32 ± 17	0.004
Need of invasive respiratory support, no. (%)	55 (49)	29 (59)^a^	15 (58)^a^	11 (30)	<0.0001
Duration of mechanical ventilation, d	4 ± 4	5 ± 4^a^	4 ± 2^a^	2 ± 2	<0.001
Need of vasoactive drugs, no. (%)	40 (36)	23 (47)^a^	11 (42)	6 (16)	0.009
Duration with vasoactive drugs, d	3 ± 3	3 ± 4^a^	3 ± 2^a^	1 ± 1	0.02
Renal failure during ICU stay, no. (%)	19 (17)	10 (20)	6 (23)	3 (8)	0.21
ICU stay, d	6 ± 9	7 ± 7	6 ± 5	5 ± 13^d^	0.76
Hospital stay, d	39 ± 23	42 ± 26	36 ± 19	37 ± 22	0.66
ICU mortality, no. (%)	4 (4)	3 (6)	1 (4)	0 (0)	0.32
Hospital mortality, no. (%)	5 (5)	4 (8)	1 (4)	0 (0)	0.19
60 days mortality, no. (%)	5 (5)^e^	4 (8)^e^	1 (4)	0 (0)	0.19
Crystalloids, mL/24 h	3596 ± 1261	3434 ± 964	3318 ± 1348	3888 ± 1460	0.08
Colloids^c^, mL/24 h	541 ± 608	442 ± 495	613 ± 569	623 ± 754	0.12
Rbc transfusion rate, units/24 h	2 ± 4	2 ± 1^a^	2 ± 2	4 ± 7	0.03
Fresh frozen plasma transfusion rate, units/24 h	1 ± 3	1 ± 1	1 ± 2	2 ± 5	0.11

Data are mean ± SD unless otherwise specified. The overall significance level is shown (ANOVA)

^a^ *P* < .05 compared with patients with no AHF/ARDS (Tukey test)

^b^ *P* < .05 compared with patients with ARDS (Tukey test)

^c^Synthetic colloids and albumin

^d^One patient treated in the Neuro-intensive care unit for 81 days

^e^One patient was in palliative care before the trauma

**Fig. 2:**
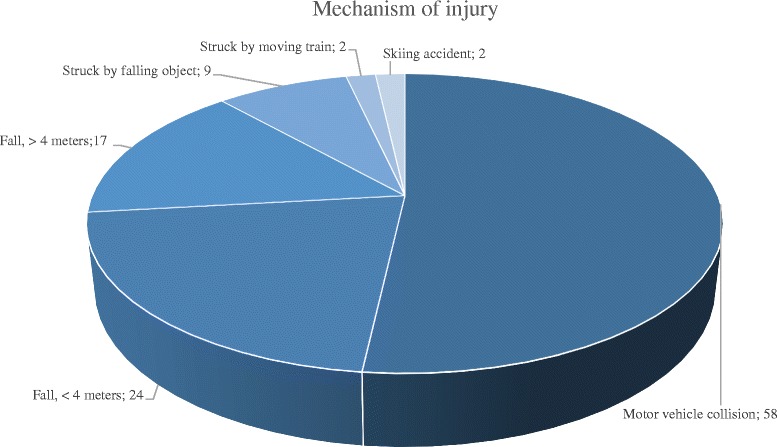
Mechanism of injury. The numbers depict number of patients per category (*n* = 112)

The mechanism of injury is presented in Fig. 2. Motor-vehicle accidents were the most common cause followed by falls. In 16 patients (15 falls and 1 motor vehicle accident), the trauma was related to a suicide attempt.

### AHF/ARDS

The total incidence of AHF/ARDS was 67 % (75/112 patients), i.e., the percentage of patients that at any period during the ICU stay fulfilled the AHF/ARDS criteria, and the allocation to the two groups was done according to the analyses of the chest images.

#### Analysis of the chest images

One hundred and forty-eight chest images were analyzed (87 images pre- (78 % of the patients) and 61 post-operative (54 % of the patients). There was a lack of consensus in 17 images, 8 pre- (9 % of the analyzed images) and 9 (15 % of the analyzed images) post-operative. After a second analysis consensus was found in the remaining 17 images. Chest images were divided by 102 chest radiograms and 46 CT-scans. The radiological examinations were performed 2 ± 3 days before surgery and 2 ± 3 days after surgery. In 12 patients with AHF pre-operative and 20 patients with AHF post-operative there was no chest images available.

#### Incidence of AHF

The total incidence of AHF was 44 % (49/112 patients). 35 patients (31 %) had AHF before surgery (14 patients with mild AHF, 18 patients with moderate AHF and 3 patients with severe AHF); 39 patients (35 %) had AHF after surgery (13 patients with mild AHF, 21 patients with moderate AHF and 5 patients with severe AHF); and 25 patients (22 %) had AHF both before and after the surgical intervention.

#### Incidence of ARDS

The total incidence of ARDS was 23 % (26/112 patients). 12 patients (11 %) had ARDS before surgery (4 patients with mild ARDS, 7 patients with moderate ARDS and 1 patient with severe ARDS); 20 patients (18 %) had ARDS after surgery (4 patients with mild ARDS, 15 patients with moderate ARDS and 1 patient with severe ARDS) and 6 patients (5 %) had ARDS both before and after surgery.

**Fig. 3:**
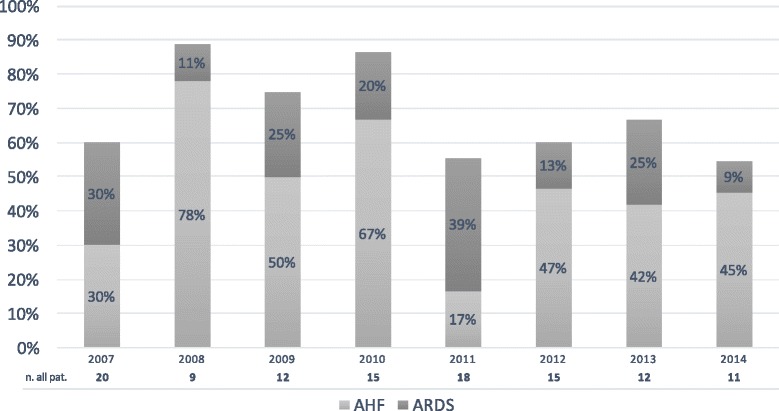
Incidence of acute hypoxic failure (AHF) and acute respiratory distress syndrome (ARDS) per year 2007–2014. n all pat. = total number of patients

There was no major change in the yearly incidence of AHF/ARDS in patients with ICU requiring pelvis fractures from 2007 to 2014 (Fig. 3).

#### Characteristics of patients with and without AHF/ARDS

In Table 1, the patients are presented in three groups; two groups where the patients had AHF or ARDS at any time during the ICU stay and one group which never developed AHF or ARDS. There were no differences in severity scores (ISS, NISS) between patients with or without AHF/ARDS. As expected, SOFA were higher in patients with AHF/ARDS than in the other patients, since the PaO_2_/FiO_2_ ratio is included in the calculation of SOFA. The patients with AHF/ARDS had more lung contusions and had been diagnosed more frequently with pneumonia than the other patients had. This resulted in more need of invasive mechanical ventilation as well as longer duration of mechanical ventilation. The patients with AHF/ARDS were more often treated with vasoactive agents, but there were no differences in the amount of fluid administered or blood product transfusions among the groups.

#### AHF/ARDS in relation to the surgical procedure (See Table 2)

**Table 2:** Effects of the surgical stabilization

Variable	Negative AHF/ARDS status change (*n* =23)	Positive AHF/ARDS status change (*n* =12)	*P* value
Injury severity score (ISS)	26 ± 11	32 ± 9	0.08
Pulmonary contusion, no. (%)	9 (39)	1 (8)	0.04
Pneumothorax before admission, no. (%)	10 (44)	3 (25)	0.38
Pneumonia, no. (%)	2 (9)	3 (25)	0.70
Time to surgery after injury, d	5 ± 3	10 ± 6	0.03
PEEP pre-operative, cmH2O	9 ± 6	7 ± 2	0.30
PEEP post-operative, cmH2O	8 ± 4	8 ± 1	0.99
Perioperative blood loss, mL	1058 ± 1277	1261 ± 1095	0.68
Perioperative fluid balance, mL	2573 ± 1859	2205 ± 1669	0.70
Perioperative crystalloids, mL	3140 ± 978	3755 ± 1311	0.16
Perioperative colloids^a^, mL	818 ± 737	528 ± 643	0.31
Perioperative rbc transfusion rate, units	3 ± 3	2 ± 1	0.51
Fresh frozen plasma transfusion rate, units	2 ± 2	2 ± 2	0.72

Data are mean ± SD unless otherwise specified

^a^Synthetic colloids and albumin

**Fig. 4:**
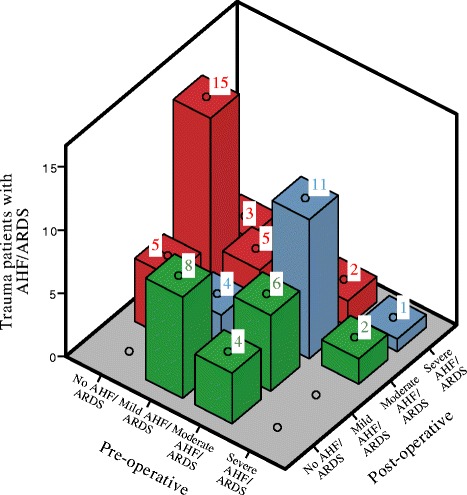
Patient with acute hypoxic failure (AHF) and acute respiratory distress syndrome (ARDS) and the individual status change pre- and post-operative. The 37 patients who did not have AHF/ARDS at any time during the ICU stay are not included in the figure. The Y-axis shows the number of patients, the x-axis the oxygenation status of the individual patients before surgery and the z-axis the oxygenation status after surgery. For example, 5 patients had no AHF/ARDS before but deveoped mild AHF/ARDS after surgery and 15 patients had no AHF/ARDS before but developed moderate AHF/ARDS after surgery. The color describes how the individual patient’s AHF/ARDS status changed post-operatively. Red = Worsen AHF/ARDS status (e.g. 15 patients had no AHF/ARDS pre-operative but had moderate AHF/ARD post-operative (the red number 15)). Blue = No AHF/ARDS status change (e.g. 11 patients had moderate AHF/ARDS pre-operative and post-operative (the blue number 11)). Green = Better AHF/ARDS status (e.g. 2 patients had severe AHF/ARDS pre-operative but had moderate AHF/ARDS post-operative (the green number 2))

There were no significant differences in PaO_2_/FiO_2_ ratio from before to after surgery (28 ± 15 kPa vs 27 ± 12 kPa). However, 23 patients with pre-operative normal lung status developed AHF/ARDS in relation to the surgical procedure, whereas 12 patients with AHF/ARDS normalized their lung condition (Fig. 4). The injury scores and the amount of fluid administrated and blood product transfused were similar in the two categories. However, the patients who developed AHF/ARDS had a higher incidence of lung contusion (*P* = 0.04). In addition, surgical stabilization was performed earlier (5 versus 10 days) in these patients (*P* = 0.03).

The individual changes of the patients AHF/ARDS status pre- versus post-operatively are displayed in Fig. 4.

#### Mortality

The ICU and hospital mortality was low. In the AHF group 8 % died (4/49 patients), in the ARDS group 4 % (1/26 patient) while in the group without AHF/ARDS none (0/37) died during the hospital stay. The deaths were not related to respiratory failure; one of the patients that died in the ICU was admitted post-operative after a cardiac arrest in the orthopedics ward (year 2010), one patient died due to sepsis (year 2007), one patient due to cerebral herniation (year 2007) and one patient died due to multi organ failure (year 2011). One patient that died within the first 60 days of admission was treated with palliative care before the trauma. No patient has died since 2011.

## Discussion

In this retrospective study analyzing 112 patients treated in a referral center ICU of a cohort of 669 patients with pelvis fractures, we found that 1) 67 % of the ICU treated patients had severe respiratory failure, 2) 23 of the ICU treated patients developed severe respiratory failure during the surgical stabilization and 3) the respiratory failure did not contribute to mortality.

The database analyzed contains all patients with pelvis fractures referred to our center. Thus, we consider that the database used is complete. However, the data regarding respiratory complications including blood gas analyses, chest radiography and ventilator settings were retrospectively collected and documented in our files. Therefore, the oxygenation parameters were not always obtained at the exact same time point as the chest radiograms. Moreover, in some instances, chest radiography was not performed and therefore it was not possible to diagnose whether the patient had ARDS. Furthermore, if we had performed more CT-scans that have a higher diagnostic sensitivity and specificity would also have modified the number of ARDS diagnosed in this study. Hence, although the total number of severe respiratory failure is correct, some of the patients now diagnosed as AHF could therefore have ARDS. In this context, it is important to recognize that one-sided pulmonary infiltrates are not consistent with a diagnosis of ARDS, and in fact, many of the patients had one-sided lung changes.

Compared with other studies the incidence of respiratory failure including ARDS in patients with pelvis fractures may seem high. However, we analyzed only the patients treated in the ICU. If one uses as denominator the whole cohort of patients referred or treated at our hospital, the incidence of severe respiratory failure will be 11 % and of ARDS 4 %. These numbers agree with other studies in unselected material that have reported an incidence of ARDS between 1.5 and 23 % [10, 16, 17].

We found that lung contusion was associated with a development of respiratory failure in relation to the surgical stabilization. Although our results cannot pinpoint whether the deterioration in lung function occurred during of after surgery, this finding is in line with Pape et al. [18] who found that nailing of femur fractures in patients with severe chest trauma was associated with a 33 % incidence of ARDS. Likewise, Hoyt et al. [19] found a higher incidence of ARDS in patients with chest trauma (20 % compared with 3.3 %). We think that it is important to consider not only the surgical procedure that can be “a second hit” and deteriorate the lung function. In addition, anesthesia and the ventilatory management are important factors in this context. In fact, it is found that inappropriate ventilation during surgery increases post-operative respiratory complications [20]. In neither of these studies, it is reported whether lung protective ventilation was used. However, Schreiter et al. [21] have shown that lung complications in patients with lung contusion can be reduced with protective ventilation including low tidal volumes, lung recruitment and adequate PEEP. Also, in our study lung recruitment maneuvers or adequately high PEEP were not used routinely. Therefore, we believe that a protective ventilatory management during the surgical stabilization could have reduced the high incidence of new respiratory failure in our study.

In contrast to most other studies [9, 10, 16, 17], early surgical stabilization was associated with development of new respiratory failure. This finding must be interpreted with caution; except what we discussed above regarding the higher incidence of lung contusion in these patients, this finding can be due only by chance since we did not a priori have this as an outcome parameter. Furthermore, it could be so that respiratory effect of lung contusion developed by time independent of the surgical procedure. In the patients, who were operated on later, the lung function did not in most cases deteriorate and even in many incidences improve. The latter finding could be due to that the orthopedic surgeon might have delayed the surgical stabilization because of an initial serious lung condition until the lungs had improved. Thus, whether the same results had been obtained if the surgery had been performed earlier is unknown.

We found that the mortality rate is low in patients with pelvis fractures in agreement with other studies [16, 17]. Although there were five patients who died in the AHF/ARDS group compared with none in the group without respiratory failure, in no case it could be found that the respiratory condition was a clear contributing factor. This finding agree with Treggiari et al. [2], who found, that after adjusting for age, ISS and acute physiological score (APS) there was no association of mortality with ARDS in critically ill trauma patients. Thus, we consider that the respiratory failure is manageable and is not an important cause of death in this patient category.

### Limitations

Our study has many limitations; 1) it is a retrospective study and the data e.g. blood gases and chest radiographs were not obtained at the same time point in all patients, 2) the incidence of ARDS may be higher than what we reported since chest radiography was not performed in every patient, 3) it is an observational study and since blood gases were not sampled immediately in relation to surgery, the effect of surgery and particularly any difference between early and late surgical stabilization should be interpreted cautiously 4) the material is limited and only data from patients treated in the ICU was analyzed. The mortality rate of the other patients was therefore not assessed.

## Conclusion

In conclusion, we found that the incidence of acute respiratory failure in ICU treated patients with pelvis fractures was high and that overall no significant change in lung function in occurred in relation to surgical stabilization. However, in patients with lung contusion this study indicated that surgical stabilization was associated with a deterioration in lung function. Furthermore, we found, that mortality was low and was probably not related to the respiratory condition.

### Ethics, consent and permissions

All patients gave their consent before inclusion in the database. No individual data are presented.

## Abbreviations

AHF: acute hypoxic failure
AIS: abbreviated injury scale
APS: acute physiological score
ARDS: acute respiratory distress syndrome
CT: computed tomography
FiO_2_: inspired oxygen fraction (FiO_2_)
GCS: glasgow coma scale
ICU: intensive care unit
ISS: injury severity score
NISS: new injury severity score
PaO_2_: arterial oxygen tension
PaO_2_/FiO_2_: arterial oxygen tension/Inspired oxygen fraction
PEEP: positive end expiratory pressure
Rbc: red blood cell concentrate
SAPS 3: simplified acute physiology score
SD: standard deviation
SOFA: sequential organ failure assessment score
